# *Eggerthella lenta* bacteremia successfully treated with ceftizoxime: case report and review of the literature

**DOI:** 10.1186/s40001-021-00582-y

**Published:** 2021-09-20

**Authors:** Shuming Jiang, Jianfei E, Dengchao Wang, Yanjiao Zou, Xiao Liu, Hualiang Xiao, Yuan Wen, Zongyao Chen

**Affiliations:** 1 Pathology Department, People’s Hospital of Deyang City, Deyang, China; 2 Department of Clinical Laboratory, People’s Hospital of Deyang City, Deyang, China

**Keywords:** *Eggerthella lenta*, Bacteremia, Ceftizoxime, Appendicitis

## Abstract

*Eggerthella lenta* is a normal human microflora that is anaerobic, non-sporulating, and Gram positive. However, an increasing number of studies have shown that it could also be an important pathogen for humans, even causing life-threatening infection under certain conditions. However, understanding its pathogenic mechanism and treatment options still need to be improved; more clinical data are needed to explore it further. In this article, we report a case of ceftizoxime-cured *E. lenta* bacteremia and review the recent literature to provide more clinical data for the diagnosis of *E. lenta* bacteremia. Our report suggests that the frequency of *E. lenta* bacteremia is increased in patients with hematologic or solid organ cancer, diabetes mellitus and also in those with appendicitis.

## Introduction

*Eggerthella lenta* was first described by Arnold Eggerth in 1935 [1], which was originally named *Eubacterium lentum*. *E. lenta* is a normal bacterium that can colonize the human gut, female reproductive tract, oral cavity, and prostate gland [2]. Due to the nature of strictly anaerobic and slow growth, *E. lenta* is difficult to culture and identify. Although, until recently, several microbial identification techniques, particularly mass spectrometry, were used to identify bacteria [3, 4], an increasing number of *E. lenta* infections in humans have been reported, referring to bloodstream infection [5–7], liver abscess [8], bacterial vaginosis [9] and meningitis [10]. However, a unified standard treatment and its pathogenic mechanism have not yet been recognized. Here, we report cases of *E. lenta* bacteremia cured by ceftizoxime and review the existing recent literature. This article aims to add to existing data for the timely diagnosis and treatment of *E. lenta* bacteremia.

## Materials and methods

### Case presentation

A 32-year-old man was admitted to our hospital with metastatic right lower abdominal pain for more than 20 h. He had a healthy history. On admission, he had fever (39.8 ℃), laboratory tests showed elevated C-reactive protein (127 mg/L), elevated white blood cell count (20.67 × 10^9^/mL with 87.3% neutrophils), elevated procalcitonin (0.95 ng/mL) and normal biochemical and coagulation parameters. Color Doppler ultrasound suggested appendicitis. Two sets of blood cultures (four blood culture bottles) were consecutively taken and incubated for pathogenic detection before the use of ceftizoxime (3 g, ivgtt, q8h) in empirical anti-infective therapy. The patient underwent an appendectomy. The patient’s temperature and white blood cell count gradually dropped to normal 2 days later, but procalcitonin was still abnormal (0.35 ng/mL).

Blood culture (two anaerobic bottles) appeared positive in the BacT/ALERT 3D blood culture system (bioMerieux, France) for 49.8 and 56.0 h, respectively. Furthermore, positive broth culture was incubated in an anaerobic airbag (bioMerieux, Lyon, France) for 72 h, and small transparent gray colonies can be seen. The isolated strain was identified as *Eggerthella lenta* with the help of MALDI-TOF (Bruker Daltonik GmbH, Germany), with a value of 2.08, indicating a high confidence result. Additionally, 16S rRNA sequencing was performed to further confirm the precision of our identification. The result showed that our strain had the highest identity with the *Eggerthella lenta* DSM 2243 strain. The result of 16S rRNA sequencing was submitted to the NCBI database with accession number MW295842.

From the results of the microbiological examination, the clinicians believed that the current antibacterial agent (ceftizoxime) is effective and should not be replaced. Seven days after surgery, the patient recovered and was discharged from the hospital.

### Literature search

We searched the literature in PUBMED from 2000 to 2020, using the search terms “*eggerthella lenta and bacteremia” or “eggerthella lenta and blood”.* From the retrieved literature, we selected the literature with a detailed description of the case and summarized the patient's clinical characteristics.

## Discussion

In recent years, *Eggerthella lenta* has received increasing attention as a human pathogen. However, due to its fastidious nature, some cases of *E. lenta* infection could previously have been undiagnosed by conventional biochemical methods [11]. Methods such as 16S ribosomal RNA (16S rRNA) gene sequencing techniques were considered the most accurate method. However, this method is costly and may not be readily available. Newer methods, such as MALDI-TOF MS, can quickly identify *E. lenta* and are more readily available at relatively low cost [4].

Several reports have shown that *E. lenta* can be isolated from humans at multiple sites [12–15], which presented a greater challenge for its clinical diagnosis and treatment. Recent research has attempted to summarize the determinants of *E. lenta* bacteremia. An earlier study showed that most *E. lenta* bacteremia exhibited serious intra-abdominal pathology [16]. Our case, the patient admitted to the hospital due to appendicitis, was also consistent with this observation. *E. lenta,* which colonizes the intestinal tract, could easily invade the bloodstream through the damaged mucosa and cause bacteremia. However, other studies showed that patients with cancer, decubitus ulcers, and diabetes mellitus were more likely to suffer from *E. lenta* bacteremia [17, 18]. All of these investigations showed different results due to the different populations involved.

We summarized 175 patients with *E. lenta* bacteremia from the literature [5–7, 10, 19–31], along with the case reported here. The clinical characteristics of these 176 cases in total are presented in Table 1. Males (102/176) and individuals around 60 years of age (mean age = 61.2) were highly likely to be infected with *E. lenta*, while 63.3% had fever, 46.7% abdominal pain, 27.8% vomiting, and 16.1% diarrhea. This suggests that about 36.7% of the patients did not show fever symptoms in the case of *E. lenta* bacteremia, making it a great challenge for clinicians to accurately diagnose the bloodstream infection caused by this bacillus. Our data showed that the most common underlying health conditions for *E. lenta* bacteremia were solid or hematologic organ cancer (31.1%), diabetes mellitus (25.6%), and cardiovascular diseases (15.0%). These three conditions represented 71.7% of the total of 176 patients, while the main sources of infection in all of these patients were the gastrointestinal tract (65.0%), skin and soft tissues (19.4%), and abscess (8.9%). Of all initial symptoms, appendicitis accounts for the highest proportion, 21.8%, much higher than colitis, the second largest, 9.4%. Appendicitis is often accompanied by perforation or even peritonitis. *E. lenta,* normally colonized in the gastrointestinal tract, could invade the bloodstream more easily. Therefore, patients with tumors, diabetes mellitus, and appendicitis should pay more attention to bloodstream infection by *E. lenta*.

**Table 1:** Clinical features of 176 patients with *E. lenta* bacteremia

Characteristic	Number of patients	Percentage (%)
Male/female	102/74	–
Mean age, year	61.6 (19–86)	–
Presenting symptoms at admission		
Fever	114	63.3
Abdominal pain	84	46.7
Emesis	50	27.8
Diarrhea	29	16.1
Underlying health status		
Hematologic or solid organ cancer	56	31.1
Diabetes mellitus	46	25.6
Cardiovascular diseases	27	15.0
Chronic kidney disease	19	10.6
Dementia	15	8.3
Chronic pulmonary disease	13	7.2
Peripheral vascular disease	10	5.6
Cerebral vascular disease	9	5.0
Liver cirrhosis	7	3.9
None	7	3.9
HIV infection	6	3.3
Rheumatologic disease	4	2.2
Crohn’s disease	1	0.6
Likely source of infection		
Gastrointestinal	117	65.0
Appendicitis	39	21.7
Colitis	17	9.4
Diverticulitis	16	8.9
Small bowel obstruction	9	5.0
Colon cancer	7	3.9
Gastrointestinal bleeding	3	1.7
Other	9	5.0
Skin and soft tissues	35	19.4
Decubitus ulcer	9	5.0
Sacral ulcers	9	5.0
Other	17	9.4
Abscess	16	8.9
Intra-abdominal abscess	10	5.6
Liver abscess	3	1.7
Other	3	1.7
Pulmonary	2	1.1
Pancreatitis	1	0.6
Blunt trauma	2	1.1
Bacteremia caused by an unknown source	24	13.3

The most efficient antibacterial drugs against *E. lenta* infection are metronidazole, amoxicillin–clavulanate, and carbapenems; it is resistant to ceftriaxone [7, 16, 18, 20, 32]. Our patients empirically used ceftizoxime without clear pathogenic bacteria and showed good efficacy, which to our knowledge, has not been reported so far in the literature. Although both ceftizoxime and cefotaxime belong to the third generation of cephalosporins, ceftizoxime has a higher antianaerobic activity than cefotaxime [33], which may be the reason why our patients have a better response to ceftizoxime. However, there could be other reasons. An earlier study divided 29 patients infected with *E. lenta* into two groups and found one susceptible to beta-lactam drugs, while the other was completely resistant to beta-lactam antibiotics [34]. From this, we could speculate that our strain belonged to the group that was sensitive to beta-lactam antibiotics. It was unfortunate that we were unable to perform antimicrobial susceptibility tests due to the limitations of our conditions. Although more tangible evidence is needed to confirm, our findings also contribute to a better understanding of the resistance characteristics of *E. lenta*.

To conclude, the frequency of *E. lenta* bacteremia is increased in patients with hematologic or solid organ cancer, diabetes mellitus and also in those with appendicitis. *E. lenta* bacteremia in our study was successfully treated with ceftizoxime; however, this needs to be further confirmed in future research.

## Data Availability

Not applicable.
